# Genetic and Immunologic Interactions Driving STAT3 Signaling and Macrophage Polarization in Acute Myeloid Leukemia

**DOI:** 10.3390/ijms27208957

**Published:** 2026-10-09

**Authors:** Kvan Omar Ahmed, Measer Abdullah Ahmed, Goran Othman, Shukur Wasman Smail

**Affiliations:** 1 Department of Biology, College of Education for Pure Science, Tikrit University, Tikrit 34001, Iraq; dr.measerahmed@tu.edu.iq; 2 Department of Medical Microbiology, College of Medicine, University of Kirkuk, Kirkuk 36001, Iraq; 3 Department of Medical Laboratory Technology, Erbil Health and Medical Technical College, Erbil Polytechnic University, Erbil 44001, Kurdistan Region, Iraq; goran.othman@epu.edu.iq; 4 Department of Biology, College of Science, Al-Qalam University, Kirkuk 36001, Iraq; 5 College of Pharmacy, Cihan University-Erbil, Erbil 44001, Kurdistan Region, Iraq; 6 Department of Biology, College of Science, Salahaddin University-Erbil, Erbil 44001, Kurdistan Region, Iraq; 7 Department of Medical Science, Respiratory Medicine, and Allergology, Uppsala University and University Hospital, 75185 Uppsala, Sweden

**Keywords:** acute myeloid leukemia, CCAAT/enhancer-binding protein alpha, runt-related transcription factor 1, signal transducer and activator of transcription 3 signaling, oncostatin M, tumor-associated macrophages, inflammatory cytokines

## Abstract

Acute myeloid leukemia (AML) is an increasingly recognized malignancy sustained by a dysregulated inflammatory niche, in which genetic alterations and microenvironmental signaling converge to drive pathogenesis. This review synthesizes emerging evidence linking genetic mutations and immune dysregulation in AML, proposing an integrated mutation-macrophage-signal transducer and activator of transcription 3 (STAT3) axis as a conceptual framework of disease progression. It proposes that mutations in CCAAT/enhancer-binding protein alpha (*CEBPA*) and Runt-related transcription factor 1 (*RUNX1*), despite conferring divergent genetic risk, converge on a common pathway of inflammatory cytokine overproduction, including interleukin-1 beta (IL-1β), tumor necrosis factor alpha (TNF-α), interleukin-6 (IL-6), and interleukin-10 (IL-10), as well as C-C motif chemokine ligand 2 (CCL2)-mediated recruitment of tumor-associated macrophages. These educated macrophages are proposed to undergo M2-like polarization, releasing oncostatin M (OSM) and IL-6, which are positioned to activate the Janus kinase/signal transducer and activator of transcription 3 (JAK/STAT3) survival pathway in leukemic blasts. Concurrently, IL-10 produced by regulatory T cells, stromal cells, and M2-like polarized macrophages within the leukemic niche signals through its cognate receptor to potently activate STAT3, suppressing antigen-presenting cell function, downregulating major histocompatibility complex class II (MHC class II) expression, and impairing effective anti-tumor T-cell immunity, thereby potentially reinforcing the immunosuppressive architecture that shields leukemic blasts from immune surveillance. This proposed feed-forward loop is associated with transcriptional upregulation of the anti-apoptotic factor BCL2-like 1 (*BCL2L1*) and the negative-feedback regulator suppressor of cytokine signaling 3 (*SOCS3*), a pattern consistent with chemoresistance and impaired negative-feedback control of STAT3 signaling. Translational biomarkers, including OSM, IL-6, IL-10, and *SOCS3*/*BCL2L1* transcripts, may quantify this axis, offering a dynamic, functional complement to static genetic risk stratification. Ultimately, this proposed triad suggests that aberrant inflammation may represent a shared therapeutic vulnerability; targeting the IL-6/oncostatin M receptor (OSMR)/STAT3 signaling nodes or reprogramming macrophage polarization could, if validated, disrupt this circuit in selected mutational subtypes.

## 1. Introduction

Acute myeloid leukemia (AML) is an aggressive heterogeneous malignant clonal disorder characterized by uncontrolled myeloid blast (progenitor cell) proliferation within the bone marrow accompanied by a block in differentiation that impairs effective hematopoiesis, resulting in clinically significant cytopenia [1]. AML incidence rises with age. The median age at diagnosis is 68 years, and two-thirds of cases occur in patients older than 55 years [2]. Leukemia ranks 13th in incidence among cancers worldwide, with an estimated 487,000 new cases in 2022, and 10th in cancer-related mortality, with an estimated 305,000 deaths [3]. Long-term burden analysis from 1990 to 2021 demonstrates an 82.25% increase in global leukemia incidence over this period [4]. Overall, five-year survival in AML is approximately 31.9%, decreasing to 11.2% among patients aged >65 years. These outcomes revealed a disproportionate burden of this disease in older adults [5].

AML constitutes a heterogeneous hematologic malignancy with multifactorial pathogenesis which includes accumulated mutations, genome instability, immune dysregulation, epigenetic reprogramming, resistance to apoptosis by various mechanisms, disruption of metabolic pathways, sustained proliferative signaling, activation of inflammatory cascades and immunosuppression of bone marrow microenvironment [6]. AML originates within the bone marrow and then blasts may enter the peripheral bloodstream. It is found to reach other parts of the body such as lymph nodes, spleen and some other organs. The term “acute” reflects the aggressive course of the disease, which arises from genetic and epigenetic alterations in hematopoietic stem cells (HSCs) and their myeloid progenitors; these generate immature white blood cells (blasts) that proliferate abnormally and become malignant leukemic cells within the bone marrow microenvironment (BMM) [7].

Recurring AML-associated mutations accumulate progressively in hematopoietic progenitor cells with age driving clonal expansion but without leukemic development, known as clonal hematopoiesis of indeterminate potential (CHIP) phenomenon [8]. Notably, clonal hematopoiesis progresses to overt AML at an estimated rate of approximately 0.5–1% per year [9]. Signal transducer and activator of transcription 3 (STAT3) signaling has been implicated in the protective bone marrow microenvironment of AML and in blast survival, chemoresistance and immune evasion. STAT3 is constitutively activated in many, though not all, leukemia subtypes and has been proposed as an oncogenic driver. Its sustained activation has been linked to leukemic cell survival, proliferation and resistance to apoptosis, and is frequently associated with poor prognosis; targeting STAT3 is therefore under investigation as a strategy to overcome resistance and improve outcomes in AML [10].

The present investigation aims to integrate current evidence into a cohesive model of AML pathogenesis, emphasizing the interrelationship between specific genetic mutations and the immunologic tumor microenvironment. We will critically examine the proposed biologic mechanisms through which mutations in the CCAAT/enhancer-binding protein alpha (*CEBPA*) and runt-related transcription factor 1 (*RUNX1*) genes, despite their divergent molecular functions, may converge to dysregulate inflammatory cytokine signaling. A core focus is to clarify how mutation-initiated cascades may involve the recruitment of tumor-associated macrophages (TAMs) and their M2-like polarization. Additionally, this review will examine how educated macrophages, through sustained production and release of cytokines such as oncostatin M (OSM) and interleukin-6 (IL-6), amplify oncogenic signaling through the oncostatin M receptor (OSMR)-STAT3 axis in leukemic blasts. Ultimately, by integrating molecular and immunological perspectives, this review proposes the “Mutation–Macrophage–STAT3 Triad” as a conceptual axis linking genetic alteration to immune signaling modulation.

Given that direct mechanistic evidence linking *CEBPA* and *RUNX1* mutations to macrophage polarization and STAT3 pathway activation remains limited within AML specifically, this review integrates findings derived from other hematologic malignancies and solid-tumor systems into a biologically plausible, testable conceptual model. Findings extrapolated from non-AML systems should be interpreted as hypothesis-generating rather than as evidence validated in AML. The critical contribution of this review is therefore not the identification of a previously unknown molecular interaction, but the construction of an evidence-ranked framework that separates three levels of support: (i) AML-specific clinical or experimental evidence, (ii) mechanistic evidence from non-AML systems that is biologically plausible but not yet validated in AML, and (iii) explicit hypotheses generated by integrating these levels. Unlike prior accounts of isolated TAM-STAT3 or cytokine-survival circuits, the proposed triad links mutation-defined AML subgroups to the cellular source, receiving compartment, and measurable outputs of STAT3 signaling. Its value lies in generating discriminating predictions, including whether *CEBPA*- and *RUNX1*-altered AML differ in macrophage-state composition, whether stromal OSMR rather than blast OSMR predicts pathway dependence, and whether cytokine blockade loses efficacy when parallel niche programs remain active. The model should be judged by these testable predictions rather than treated as proof of a unitary pathway.

## 2. STAT3 Signaling and the Cytokine Feedback Loop

### 2.1. Overview of STAT3 Activation via IL-6/JAK/gp130 Pathway

STAT3 belongs to a family of cytoplasmic transcription factors. The Src homology 2 (SH2) domain plays a critical role in activation and mediates receptor recruitment following tyrosine phosphorylation at Tyr705, which promotes homodimerization [11]. Consequently, the active dimer translocates to the nucleus, where its DNA-binding domain (DBD) binds specific promoter sequences to regulate genes responsible for proliferation [12].

STAT3 is primarily activated by IL-6, a member of a cytokine family released by immune cells and critical for inflammation and cell survival, through a highly regulated, multistep process initiated by the assembly of a receptor complex at the cell membrane [13]. These phospho-tyrosine motifs serve as docking sites for the SH2 domains of inactive STAT3 monomers in the cytosol. Once recruited, JAKs phosphorylate STAT3 at the critical tyrosine residue Tyr705. The phospho-Tyr705 of one STAT3 molecule engages the SH2 domain of another STAT3 molecule, generating a stable homodimer complex [14]. The phosphorylated dimer subsequently translocates into the nucleus, where it binds to gamma-activated sequences at the promoter of target genes, resulting in transcription of genes that contribute to cell proliferation and survival in lung adenocarcinoma [15].

### 2.2. Downstream Transcriptional Targets: SOCS3 and BCL2L1

STAT3 directly regulates the expression of several genes critical for cancer cell fate. Among these, *BCL2-like 1 (BCL2L1)* (encoding Bcl-xL) is a well-established anti-apoptotic factor. STAT3-mediated upregulation of *BCL2L1* enhances tumor cell survival by inhibiting programmed cell death, contributing to resistance against chemotherapy and other stressors. Suppressor of cytokine signaling 3 (*SOCS3*), another STAT3 target, acts as a negative feedback regulator of cytokine signaling. In the tumor context, however, this feedback loop can become functionally insufficient, so that *SOCS3* upregulation may be more appropriately regarded as an indicator of dysregulated or impaired pathway control rather than *SOCS3* itself acting as a direct oncogenic driver [16].

SOCS3 represents an essential mediator within the SOCS family that enforces negative feedback on the Janus kinase (JAK)/STAT axis, a function demonstrated in contexts including human immunodeficiency virus (HIV)-infected cell models, where SOCS3 dampens cytokine-induced signal transduction [17] via SH2-domain engagement of the receptor phosphotyrosine and kinase inhibitory region (KIR)-mediated occlusion of the JAK2 substrate-binding groove [18].

### 2.3. STAT3 Upregulates BCL2L1

STAT3 directly upregulates *BCL2L1*, encoding the anti-apoptotic protein Bcl-xL, which inhibits programmed cell death and supports tumor cell survival across multiple cancer types [12]. Inhibition of STAT3 reduces Bcl-xL expression, leading to increased apoptosis and reduced tumor growth [19].

### 2.4. Functional Outcomes: Survival, Proliferation, Immune Evasion

*SOCS3* and *BCL2L1* are both transcriptional targets of the IL-6/JAK/STAT3 axis, but they play functionally opposite roles; *BCL2L1* is a direct pro-survival effector, whereas *SOCS3* is a negative-feedback regulator whose elevation is best interpreted as a marker of recent/ongoing STAT3 activation rather than an independent pathogenic driver [20]. STAT3 activation induces transcription of anti-apoptotic genes such as *BCL2L1* (Bcl-xL), myeloid cell leukemia 1 (*MCL1*), and survivin, which mediate resistance to apoptosis and enhance tumor cell survival and proliferation [12,19]. *SOCS3* regulates immune cell infiltration and tumor immune evasion by constraining cytokine-dependent signaling networks that shape myeloid cell differentiation, specifically the expansion and suppressive activity of myeloid-derived suppressor cells (MDSCs). Its downregulation amplifies IL-6-driven JAK/STAT3 signaling, promoting an immunosuppressive tumor microenvironment that accelerates malignant progression and diminishes the efficacy of immune checkpoint blockade [21].

### 2.5. Clinical Correlates of IL-6/STAT3 Activation

**Figure 1:**
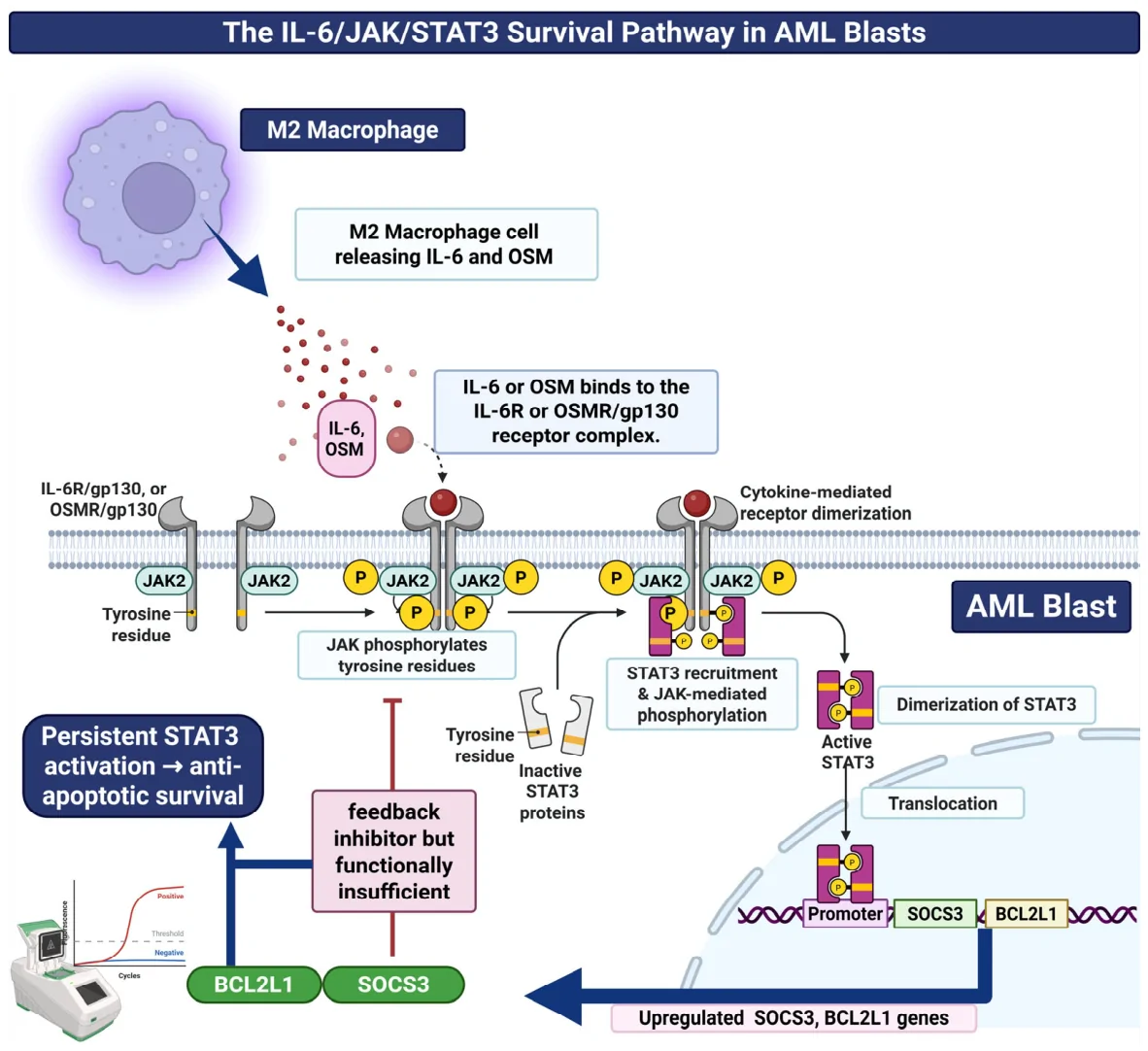
IL-6/JAK/STAT3 survival signaling in AML blasts. M2-like macrophage-associated IL-6 and OSM bind IL-6R/gp130 or OSMR/gp130 receptor complexes on leukemic blasts, inducing JAK2-mediated STAT3 phosphorylation, homodimerization, and nuclear translocation. Activated STAT3 drives transcription of *BCL2L1* (Bcl-xL; anti-apoptotic survival) and *SOCS3* (negative feedback inhibitor). Persistent *SOCS3* overexpression reflects functionally insufficient feedback attenuation, sustaining constitutive STAT3 activation and chemoresistance. *BCL2L1* transcript levels are quantified by RT-qPCR (inset). Arrows, activating signals; flat-headed lines, inhibitory interactions; P, phosphorylation. AML, acute myeloid leukemia; *BCL2L1*, B-cell lymphoma 2-like protein 1; IL-6, interleukin-6; IL-6R, IL-6 receptor; *JAK2*, Janus kinase 2; OSM, oncostatin M; OSMR, OSM receptor; RT-qPCR, reverse transcription quantitative PCR; *SOCS3*, suppressor of cytokine signaling 3; STAT3, signal transducer and activator of transcription 3. Created in BioRender. Zangana, K. (2026) https://BioRender.com/3kq206y, accessed on 21 May 2026.

Clinical evidence, largely from solid tumors, links the dysregulation of the IL-6 and STAT3 axis to aggressive tumor behavior and adverse clinical outcomes. Elevated levels of IL-6 and activated STAT3 within the tumor microenvironment serve as potent, stage-independent prognostic biomarkers, consistently associated with reduced overall survival, decreased disease-free survival, and a higher relapse rate in several solid tumors. Moreover, IL-6 overexpression in the tumor microenvironment directly promotes tumor cell proliferation and survival through activation of STAT3 in solid tumors such as oral cancer [22]. Clinically, elevated IL-6 and transcription of *STAT3* downstream targets consistently predict aggressive disease, high relapse risk, and highlight this axis as both a prognostic biomarker and a rational therapeutic target [23], as shown in Figure 1.

## 3. Macrophage Polarization and OSMR Signaling

### 3.1. M1-like vs. M2-like Macrophage Phenotypes in AML Bone Marrow

The BMM in AML constitutes a sophisticated immunological niche, within which macrophages, in addition to their various physiological and pathological roles, assume a key regulatory role in pathogenesis and immune modulation of the tumor. Macrophages within the BMM are broadly subclassified into anti-tumor (M1-like) and pro-tumor (M2-like) polarization states, with the M2-like phenotype promoting tumor progression and immune suppression within the tumor microenvironment (TME) [24]. This M1-like/M2-like phenotype dichotomy is a simplified heuristic; in vivo, macrophages occupy a continuum of context-dependent activation states rather than two fixed, mutually exclusive populations, and this terminology is used throughout the review for conceptual clarity rather than to imply discrete cellular subsets. In hepatocellular carcinoma, tumor-associated macrophages expressing high levels of programmed death-ligand 1 (PD-L1) have been shown to directly induce CD8+ T-lymphocyte exhaustion, characterized by upregulation of exhaustion markers and suppressed cytotoxic function leading to impaired immune response and reduced cytokine production [25]. Moreover, M2-like polarized macrophages release IL-10 and TGF-β, which attenuate cytotoxic T cells and promote regulatory T-cell differentiation [26].

Importantly, AML-associated macrophages cannot be reduced to a binary M1-like/M2-like switch. Single-cell analyses identify multiple macrophage populations and activation trajectories, including Fc fragment of IgA receptor (FCAR)-positive and Fc gamma receptor IIIA (FCGR3A)-positive states enriched in patients who fail to achieve complete remission, with distinct metabolic and intercellular-communication programs [27]. These states may coexist within the same marrow and change after chemotherapy, reflecting recruitment, local differentiation, and reversible niche education rather than fixed terminal polarization. Spatial position is also likely to matter: macrophages adjacent to vascular, stromal, adipocytic, or blast-rich regions may receive different combinations of IL-6, IL-10, hypoxic, and metabolic signals. Accordingly, “M1-like phenotype” and “M2-like phenotype” are retained only as broad functional descriptors; future studies should define TAMs by transcriptomic program, surface phenotype, anatomical neighborhood, and temporal state [28].

### 3.2. OSM–OSMR Signaling Promotes M2-like Immune Suppression

OSM is an IL-6 family cytokine that signals through its receptor (OSMR) and has been proposed to influence macrophage polarization, although this has not been demonstrated in AML. Upon OSM/OSMR engagement, OSMR pairs with the co-receptor gp130, initiating intracellular signaling and thereby activating JAK1/JAK2 kinases, which results in phosphorylation and translocation of STAT3 into the nucleus to upregulate its downstream target genes [29]. Activation of the OSM/OSMR/JAK/STAT3 pathway within macrophages induces transcription of genes that contribute to an M2-like phenotype, such as IL-10, arginase 1 (*ARG1*), cluster of differentiation 163 (*CD163*) and *CD206*, an effect demonstrated in glioblastoma-associated macrophages and not yet confirmed in the AML bone-marrow niche [30]. Consequently, this phenotypic remodeling may contribute to tumor progression and reduced anti-tumor immunity. Furthermore, in non-AML systems OSM/OSMR signaling has been linked to proliferation, survival and apoptosis resistance through cell cycle and anti-apoptotic genes such as *BCL2*, *MYC* and *MCL1* [31]; in AML, OSMR appears to be expressed mainly by stromal cells rather than blasts. Serum OSM and soluble OSMR have been proposed as candidate-independent biomarkers of induction response and early mortality in a retrospective AML cohort [32]. These findings suggest that the OSM-JAK/STAT3 axis may merit investigation as a therapeutic target; whether interventions can disrupt OSM-dependent signaling in AML remains to be tested.

### 3.3. Macrophage-Driven STAT3 Feed-Forward Signaling

The amplification loop operates via autocrine and paracrine signaling; IL-6 and OSM secreted by M2-like polarized macrophages bind receptor complexes on neighboring macrophages and leukemic blasts, thus activating gp130/JAK complexes and inducing STAT3 phosphorylation, dimerization and nuclear translocation. Activated STAT3 then upregulates transcriptional programs that promote additional cytokine production and expression of survival and immunomodulatory effectors [33]. An analogous STAT3/IL-10/IL-6 feedback circuit regulating macrophage phenotype has been described in a non-malignant hepatic-injury model [34]; this is cited as evidence that such a macrophage-intrinsic STAT3 feedback circuit can operate independently of infectious or malignant triggers rather than as direct evidence of this circuit within the AML niche.

### 3.4. Soluble OSMR and IL-10 as Macrophage-Activity Biomarkers

**Figure 2:**
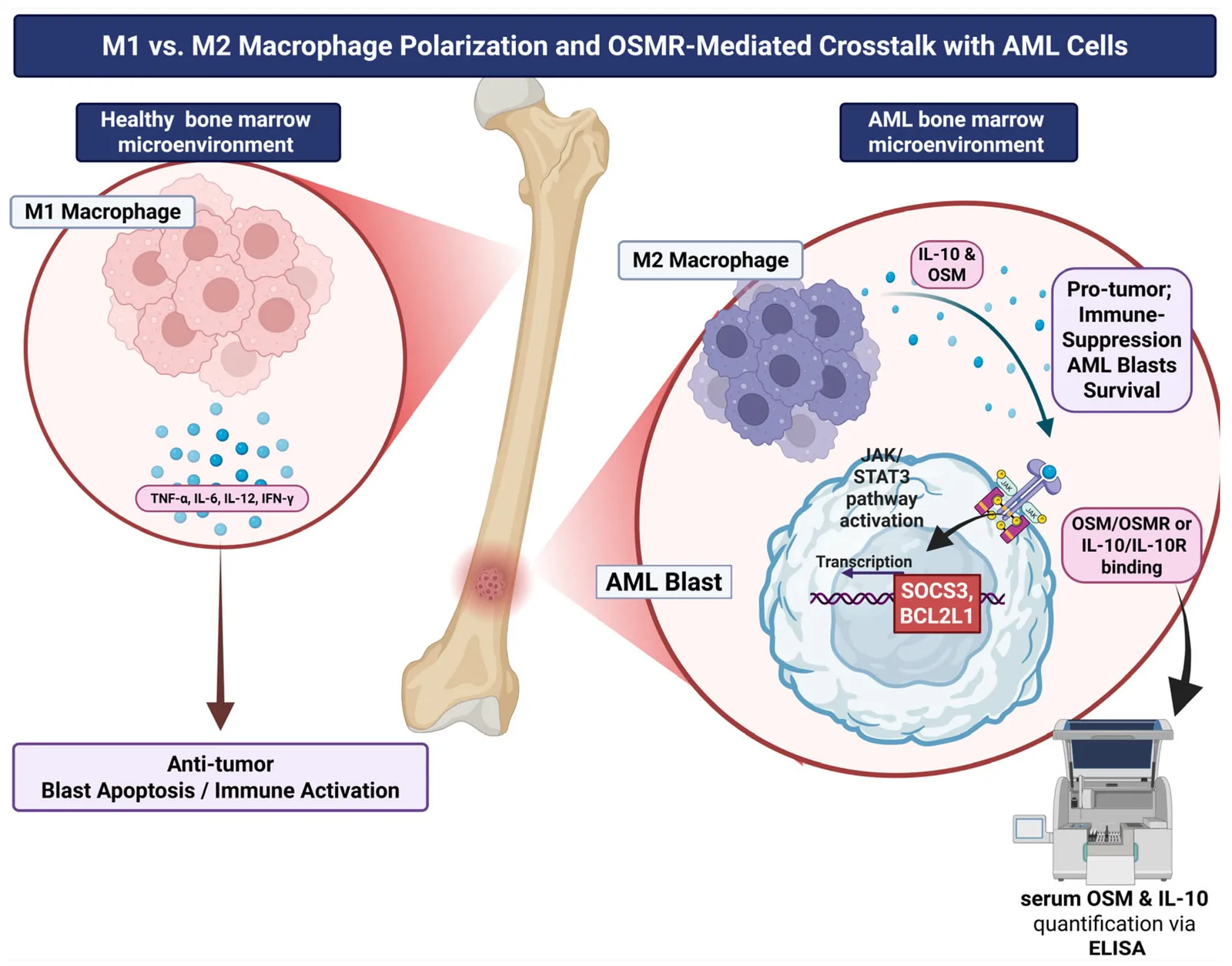
Macrophage polarization and OSMR-mediated crosstalk with AML blasts in the bone marrow microenvironment. In the healthy marrow (left), classically activated M1-like macrophages release TNF-α, IL-6, IL-12, and IFN-γ, exerting tumoricidal activity and promoting blast apoptosis. In the AML niche (right), alternatively activated M2-like macrophages secrete IL-10 and OSM, which engage OSMR/gp130 or IL-10R on leukemic blasts to activate JAK/STAT3 signaling. Nuclear STAT3 drives transcription of *SOCS3* and *BCL2L1*, sustaining blast survival, immune suppression, and chemoresistance. Serum OSM and IL-10 are quantifiable by ELISA as surrogate biomarkers of M2-like macrophage activity and STAT3 pathway engagement. Arrows, activating signals. AML, acute myeloid leukemia; *BCL2L1*, B-cell lymphoma 2-like protein 1; ELISA, enzyme-linked immunosorbent assay; IFN-γ, interferon-gamma; IL, interleukin; IL-10R, IL-10 receptor; JAK, Janus kinase; OSM, oncostatin M; OSMR, OSM receptor; *SOCS3*, suppressor of cytokine signaling 3; STAT3, signal transducer and activator of transcription 3; TNF-α, tumor necrosis factor-alpha. Created in BioRender. Zangana, K. (2026) https://BioRender.com/fwiiom3, accessed on 21 May 2026.

A soluble form of OSMR (sOSMR) has been identified and molecularly characterized [35]. Quantifying serum sOSMR via ELISA offers a means to evaluate OSM/OSMR pathway engagement, and its clinical relevance in malignancies, including AML, has subsequently been investigated [32,36]. Circulating sOSMR has been proposed to reflect OSM/sOSMR axis engagement and, indirectly, macrophage activation; its evaluation may therefore provide an indicator of immunoregulatory activity. Elevated sOSMR concentrations have been associated with adverse clinical outcomes across several malignancies, including AML [36], as shown in Figure 2.

IL-10 within the AML bone marrow niche does not act uniformly as an oncogenic signal. In regulatory T cells, stromal cells, and M2-like polarized macrophages, IL-10 reinforces an immunosuppressive microenvironment by suppressing antigen-presenting cell function and dampening cytotoxic T-cell activity via STAT3 signaling. However, several studies report that IL-10 can directly inhibit AML blast proliferation and is paradoxically associated with improved survival and remission rates in some cohorts, indicating a tumor-suppressive action on the leukemic clone itself even as it supports leukemic stem cell persistence through niche-level immunosuppression. *IL-10 receptor* (*IL-10R*) is reported to be overexpressed on AML blasts, positioning the IL-10 axis as a potential target for future immunotherapeutic strategies [37]. These findings indicate that IL-10’s net effect in AML is determined by the responding cell type and disease stage, and this should be considered when interpreting circulating IL-10 as a single biomarker of disease activity [38].

The apparently opposing clinical associations of IL-10 are therefore not necessarily mutually exclusive. High circulating IL-10 may mark an anti-inflammatory response or direct restraint of bulk blast proliferation in some disease phases, while local IL-10 signaling in macrophages, regulatory T cells, antigen-presenting cells, or leukemia stem-cell niches may still suppress immune clearance. Differences in sampling compartment, treatment phase, cellular receptor expression, AML subtype, and endpoint can consequently reverse the observed association. For this reason, total serum IL-10 should not be interpreted as a unidirectional surrogate of immunosuppression, and therapeutic inhibition would require compartment-resolved evidence that the immune-suppressive effect outweighs any blast-intrinsic growth restraint [39].

## 4. Genetic Drivers of Inflammatory Signaling

### 4.1. CEBPA Mutations

AML is a heterogeneous group of hematologic malignancies defined by arrested myeloid differentiation and clonal outgrowth of myeloid progenitor cells. Although somatic genetic alterations are major contributors to leukemogenesis, accumulating evidence implicates inflammatory signaling and cytokine network disruption as central contributors to disease initiation and progression [40]. Notably, CEBPA and RUNX1 are both pivotal regulators of myeloid differentiation and inflammatory homeostasis, and their mutations represent prominent genetic determinants of inflammatory dysregulation in AML. During granulopoiesis, *CEBPA* is expressed in HSCs and myeloid-restricted progenitors, where it induces transcription of lineage-defining genes such as those encoding granule constituents and growth-factor receptors crucial for granulocytic specification [41].

*CEBPA* encodes C/EBP*α*, a basic leucine zipper (bZIP) transcription factor that functions as a master regulator of myeloid lineage commitment and granulocytic differentiation, directing lineage specification and driving cell-cycle arrest during hematopoietic maturation. Loss-of-function *CEBPA* mutations found in AML, typically involving the N-terminal transactivation domain or C-terminal bZIP region, impair granulocytic maturation and generate progenitors that remain locked in a proliferative and undifferentiated state. Several lines of evidence indicate that these mutations also dismantle key transcriptional restraint on inflammatory gene expression. Under sustained IL-1β stimulation, HSCs initiate a C/EBPα-dependent differentiation program. However, *CEBPA*-deficient HSCs and progenitor cells fail to execute this response and instead retain stem cell-associated transcriptional programs, thereby conferring a selective growth advantage in an inflammatory microenvironment [42,43].

At the molecular level, the loss of C/EBPα removes repressive occupancy from regulatory regions of cytokine loci normally maintained in a silenced state during granulopoiesis. Loss of normal C/EBPα transcriptional control may contribute to dysregulated inflammatory gene expression through interactions with NF-κB-dependent pathways [44]. The specific effects of *CEBPA* mutation on individual cytokines and chemokines, including IL-1β, TNF-α, and CCL2, remain to be directly established in AML. At the molecular level, C/EBPα participates in the transcriptional regulation of inflammatory responses. Interactions between C/EBPα and NF-κB signaling have been implicated in inflammatory cytokine-driven myeloid differentiation [44]. Thus, disruption of normal C/EBPα function may alter inflammatory transcriptional programs in AML; however, the specific cytokine and chemokine consequences of CEBPA mutation, including effects on CCL2-mediated macrophage recruitment, require further direct experimental validation. Collectively, *CEBPA* mutations deregulate cytokine repression by eliminating CCAAT/enhancer-binding protein alpha (C/EBPα)’s transcriptional control, enabling chronic activation of inflammatory signaling networks and remodeling of the AML microenvironment. CCL2 is frequently upregulated in the leukemic microenvironment and is proposed to drive tumor-associated macrophage recruitment.

### 4.2. RUNX1 Mutations

RUNX1 is considered a master transcription factor essential for definitive hemato-poiesis and for controlling gene expression programs that direct normal blood cell development and myeloid cell functions. *RUNX1* forms a functional heterodimer with core-binding factor β (CBFβ) essential for stable DNA binding and transcriptional activity [45]. Mutations in *RUNX1* are linked to diverse hematologic disorders and myeloid cancers. Under homeostatic conditions, *RUNX1* constrains toll-like receptor-driven pathways by repressing Toll-like receptor (TLR)1/2 and TLR4 signaling outputs. This restraint is achieved through the suppression of the NF-κB branch of myeloid differentiation primary response protein 88 (MyD88) and Toll/interleukin-1 receptor domain-containing adaptor protein (TIRAP) cascade, partially mediated by its interaction with the IκB kinase (IKK) complex to stabilize IκBα and prevent its degradation. The loss of *RUNX1* disrupts this inhibitory mechanism, leading to accelerated IκBα turnover, unchecked NF-κB activation, and induction of inflammatory gene programs that are normally repressed [46].

*RUNX1* is frequently mutated in AML, and these mutations result in either loss of function or dominant-negative activity. They are characteristically associated with immature cellular morphological features and are enriched in secondary AMLs that arise from prior myelodysplastic syndromes (MDSs) [47]. These AML-associated loss-of-function and dominant-negative point mutations are molecularly distinct from the *RUNX1::RUNX1T1* fusion protein generated by t(8;21), a structurally and prognostically separate class of *RUNX1* alteration [48], and from *RUNX1* overexpression as reported in non-AML solid-tumor contexts; the three should not be treated as functionally interchangeable when interpreting downstream inflammatory or macrophage-polarization effects. *RUNX1* mutations result in derepression of pro-inflammatory cytokines, mainly TNF-α, through enhanced TLR signaling [46]. However, persistent STAT3 signaling and direct M2-like polarization via IL-6/IL-1β/STAT3 are not clearly established in myeloid cells. In solid tumors, *RUNX1* can promote M2-like polarization through alternative pathways. It is important to note that this M2-like polarizing activity has been characterized specifically in the context of *RUNX1* overexpression in solid tumors and has not been demonstrated for the loss-of-function or dominant-negative *RUNX1* alterations that predominate in AML; solid-tumor findings are presented as an illustrative alternative biological context rather than as direct evidence for the consequences of *RUNX1* loss-of-function in the AML microenvironment. *RUNX1* overexpression in solid tumors (such as colorectal cancer) can recruit and polarize macrophages toward the M2-like phenotype, mainly via CCL2 and Hedgehog pathway activation rather than direct IL-6/STAT3. *RUNX1* overexpression can elevate CCL2 as a chemokine that recruits macrophages to the tumor microenvironment and drives their M2-like polarization. In parallel, it activates the Hedgehog signaling pathway, further reinforcing M2-like macrophage skewing and tumor-promoting functions, and platelet-derived growth factor-BB (PDGF-BB) released by M2-like macrophages was found to upregulate *RUNX1* in tumor cells, establishing a feed-forward loop that sustains M2-like polarization and tumor progression [49,50].

**Figure 3:**
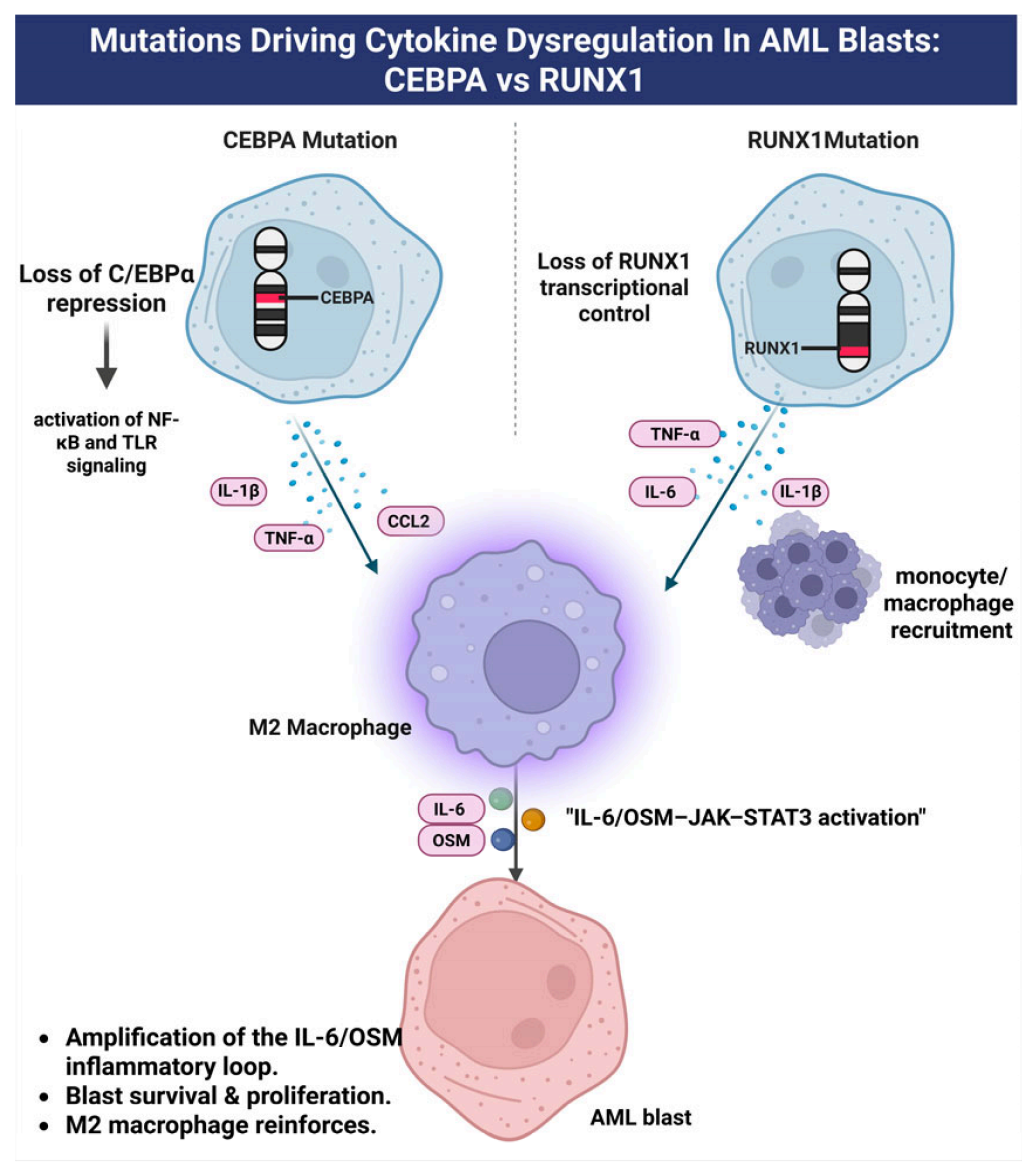
Proposed convergent cytokine dysregulation associated with *CEBPA* and *RUNX1* mutations in AML. (Left) Loss of C/EBPα transcriptional repression activates NF-κB and TLR signaling, driving overproduction of IL-1β, TNF-α, and CCL2, which recruit and polarize monocytes toward an M2-like macrophage phenotype. (Right) Loss of *RUNX1* transcriptional control derepresses TLR/NF-κB pathways, elevating IL-6, IL-1β, and TNF-α and promoting monocyte/macrophage recruitment. Despite divergent upstream mechanisms, both mutations are proposed to converge on M2-like macrophage-associated IL-6 and OSM secretion, which is hypothesized to activate the IL-6/OSM–JAK–STAT3 axis in AML blasts, amplifying the inflammatory loop and sustaining blast survival, proliferation, and M2-like polarization reinforcement. Arrows, activating signals. AML, acute myeloid leukemia; CCL2, C-C motif chemokine ligand 2; IL, interleukin; JAK, Janus kinase; NF-κB, nuclear factor kappa B; OSM, oncostatin M; STAT3, signal transducer and activator of transcription 3; TLR, toll-like receptor; TNF-α, tumor necrosis factor-alpha. Created in BioRender. Zangana, K. (2026) https://BioRender.com/psqjql5, accessed on 21 May 2026.

M2-like macrophage polarization is associated with immunosuppression and tumor progression, but the mechanism in myeloid malignancies with *RUNX1* mutation remains less clear. To date, evidence does not support sustained STAT3 activation as a direct downstream consequence of *RUNX1* mutation within myeloid malignancies. Instead, *RUNX1* loss primarily reconfigures type 1 interferon signaling networks, manifesting as augmented STAT1 and STAT2 activity rather than induction of *STAT3* transcriptional programs [51]. Although IL-6-mediated STAT3 activation drives M2-like macrophage polarization in some malignancies, this pathway has not been mechanistically linked to *RUNX1*-mutant hematopoietic compartments based on currently available data. The clinical consequences of RUNX1-driven inflammatory and immune alterations are substantial: *RUNX1*-mutant AML is characterized by unique clinicopathological attributes and poorer therapeutic outcomes. Evidence also indicates that *RUNX1* mutation defines an adverse clinical entity with reduced treatment responsiveness. In older patients and in monocytic AML (M4/M5), it supports classification as a distinct, high-risk entity requiring intensified clinical surveillance and risk-adapted therapeutic strategies. Moreover, *RUNX1* mutation in AML is associated with poorer survival outcomes, reinforcing its potential role as an adverse prognostic marker [47], as illustrated in Figure 3.

In contrast to *CEBPA* and *RUNX1*, which are proposed to influence STAT3 signaling indirectly through cytokine production and macrophage crosstalk, *JAK2* mutation activates the JAK/STAT pathway independent of upstream receptor-ligand engagement. The canonical *JAK2* V617F mutation is a rare event in de novo AML but occurs at an appreciable frequency in AML arising secondary to a pre-existing *JAK2*-mutant myeloproliferative neoplasm (MPN), where it is associated with a distinct clinicopathological profile, including higher *JAK2* V617F variant allele frequency, MPN-like megakaryocytic morphology, and a complex karyotype, relative to *JAK2*-mutant AML arising de novo [52]. Notably, even in de novo AML cohorts where *JAK2* V617F itself is uncommon, phosphorylated STAT3 is frequently detected across AML subtypes, although less often in core-binding-factor leukemias, indicating that STAT3 activation in AML is largely *JAK2*-mutation-independent and driven by alternative upstream mechanisms [53]. This observation is consistent with the paracrine, macrophage/cytokine-driven route to STAT3 activation proposed above for *CEBPA*- and *RUNX1*-mutant AML, and positions *JAK2*-mutant (predominantly secondary) AML as a cell-intrinsic, mutation-driven complement to—rather than the principal explanation for STAT3 pathway engagement across AML more broadly. Bone marrow mesenchymal stromal cells from de novo AML show broader cytokine dysregulation: elevated IL-1β, IL-6, IL-32, vascular endothelial growth factor A (VEGFA), C-X-C motif chemokine ligand 12 (CXCL12), and decreased IL-10, compared with those from secondary AML with myelodysplasia-related changes, where the increase is largely restricted to IL-6 [54]. Since IL-6 is a principal glycoprotein 130 (gp130)/JAK/STAT3 activator, this suggests secondary AML may depend on a narrower, IL-6-centered route to STAT3 engagement, while de novo AML sustains it through a broader inflammatory milieu, a hypothesis warranting direct testing with paired cytokine/STAT3-target expression data stratified by AML ontogeny.

## 5. The Mutation–Macrophage–STAT3 Triad

### 5.1. Convergent Downstream Signaling from Distinct Mutations

Mutations in transcription factors such as *CEBPA* and *RUNX1* influence myeloid differentiation; understanding the molecular mechanisms by which these distinct mutations lead to similar phenotypic outcomes provides critical insights into AML pathogenesis and therapeutic vulnerability. *CEBPA* and *RUNX1* mutations converge on the same myeloid transcriptional circuitry: RUNX1 directly controls CEBPA via promoter and distal enhancer binding, while both cooperate with purine-rich box 1 (PU.1), a transcription factor crucial for the development and differentiation of immune cells, to activate lineage-specific enhancers; mutations in either factor disrupt enhancer activation and block monocyte/macrophage differentiation [55]. *CEBPA* and *RUNX1* are cooperative master regulators of macrophage specification whose mutations converge to disrupt enhancer-dependent chromatin programs required for the monocyte–macrophage transition. CEBPA shapes the chromatin landscape, while RUNX1 controls monocytic fate and inflammatory pathways; disruption of either factor can impair macrophage maturation, alter inflammatory responsiveness, and promote leukemic persistence [56].

### 5.2. Proposed Convergence of CEBPA and RUNX1 Alterations on Macrophage-Associated STAT3 Signaling

STAT3 signaling is essential for hematopoietic stem cell self-renewal and for directing macrophage polarization, and its dysregulation is broadly implicated in AML pathogenesis [57]. Consistent with the previously described *CEBPA*- and *RUNX1*-associated cytokine profiles and TLR/NF-κB signaling mechanisms, we propose that *CEBPA* and *RUNX1* mutations converge, through distinct upstream mechanisms, on disruption of this same STAT3 signaling axis. A functional *RUNX1*-STAT3 signaling intersection, involving regulation of negative feedback regulators, has been reported in epithelial stem cell biology [58]; this is offered here only as an analogy, since neither this circuit nor any direct physical *RUNX1*-STAT3 interaction has been demonstrated in *RUNX1*-mutant AML specifically. On the other hand, mutant *CEBPA* blocks the enhancer-dependent differentiation response, producing a highly proliferative yet differentiation-arrested state that is sensitive to JAK/STAT inhibition [59]. Functionally, STAT3 has been associated with immunoregulatory macrophage polarization, including M2-like functional states [33], and its aberrant activation in AML may therefore promote leukemic self-renewal, suppress differentiation, and support tumor-permissive microenvironments [60].

We propose that the *CEBPA*-mutant leukemic niche is characterized by marked macrophage recruitment accompanied by elevated IL-6-family cytokines, particularly IL-6 and OSM, which may cooperate with IL-1β and TNF-α to reinforce inflammatory signaling in a feed-forward amplification loop. Mechanistically, engagement of the IL-6 receptor-gp130 complex is well established to activate JAKs and induce STAT3 phosphorylation at Tyr705, driving its nuclear accumulation and transcription of survival- and inflammation-associated genes [12,14]. Whether *CEBPA* loss specifically reshapes the AML microenvironment to sustain this IL-6/OSM-JAK-STAT3 signaling axis in vivo, and thereby favors a persistent inflammatory state supporting leukemic cell maintenance, has not, to our knowledge, been directly demonstrated, and should be regarded as a proposed mechanism warranting experimental validation.

IL-6 and OSM activate STAT3 in macrophages and leukemic cells via gp130/JAK signaling, sustaining an IL-6/STAT3/CEBPβ autocrine loop that drives M2-like polarization in lung adenocarcinoma [15]. CEBPA p30 mutants blunt inflammatory gene induction and cytokine secretion, but co-occurring activating *colony-stimulating factor 3 receptor (CSF3R)* mutations promote ligand-independent JAK/STAT3 activation that engages enhancers, including E2F transcription factor 2 (E2F2), to fuel proliferation. In this setting, mutant *CEBPA* sustains an enhancer-dependent differentiation-arrested leukemic state, while TAM-derived IL-6 and intercellular crosstalk further reinforce leukemic persistence, potentially increasing sensitivity to JAK/STAT pathway inhibition [59]. OSM has been reported to induce RUNX1 expression and activate STAT3 in non-AML systems, suggesting a possible feed-forward loop. Whether loss or mutation of RUNX1 perturbs this circuit in AML, and whether this produces dominant-negative-like dysregulation and OSM/G-CSF hypersensitivity, is a hypothesis proposed by this review rather than a demonstrated finding. Whether RUNX1-mutant AML is characterized by enhanced OSM/OSMR–STAT3 signaling remains unresolved. Evidence from non-AML models demonstrates that macrophage STAT3/IL-10/IL-6 feedback loops can operate [34], but whether RUNX1 mutation directly induces macrophage OSM secretion within the AML bone marrow niche has not been established.

### 5.3. Feedback Between Macrophages and Blasts Sustaining Survival Signaling

**Figure 4:**
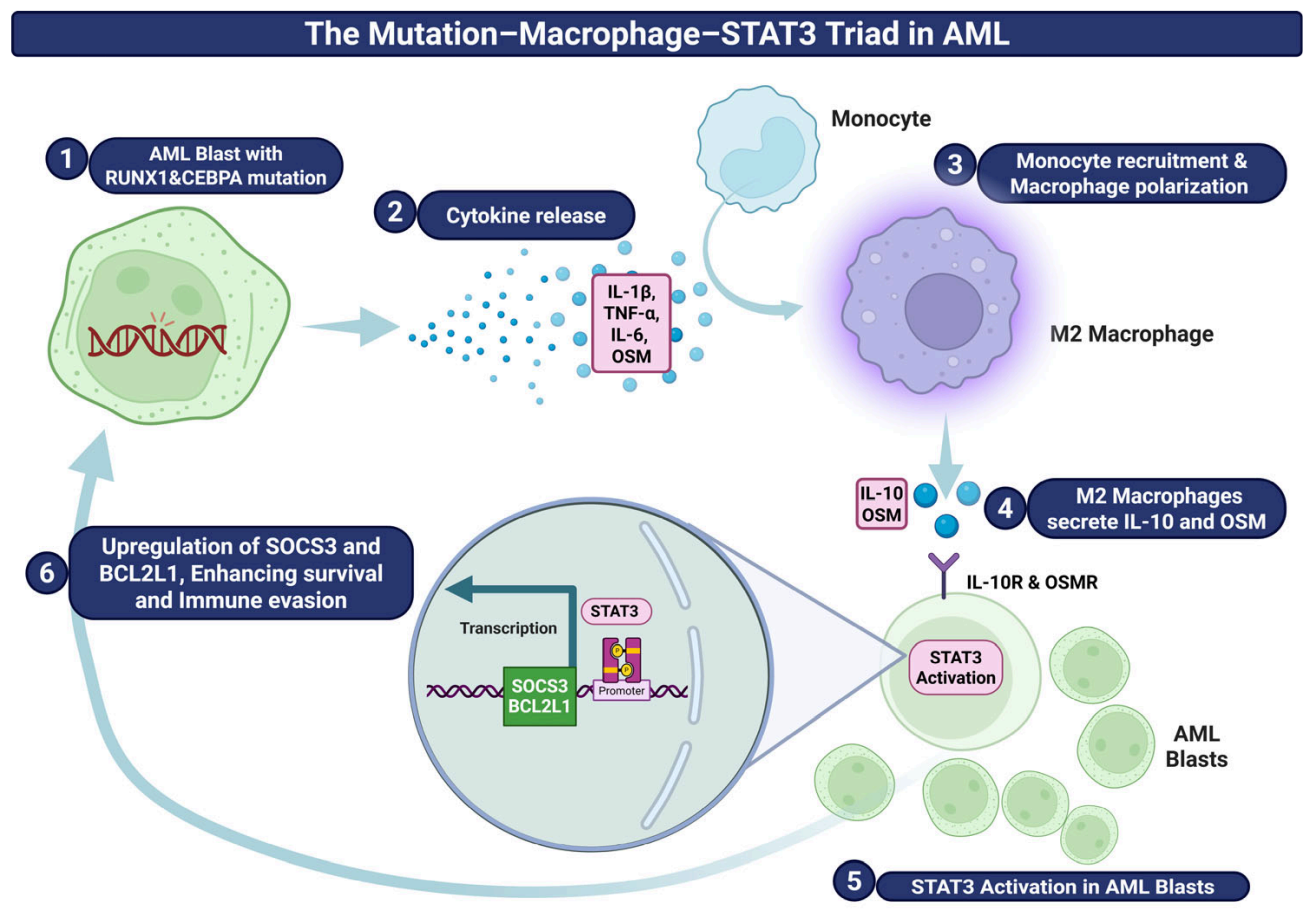
The Mutation–Macrophage–STAT3 triad as a self-reinforcing pathogenic circuit in AML. (1) In the proposed model, RUNX1- and CEBPA-mutant leukemic states are hypothesized to promote inflammatory cytokine dysregulation (2), which may contribute to CCL2-mediated monocyte recruitment and M2-like macrophage polarization (3). (4) Educated M2-like macrophages are proposed to secrete IL-10 and OSM, which would engage IL-10R and OSMR on leukemic blasts to activate STAT3 (5). Nuclear STAT3 is hypothesized to drive transcription of *SOCS3* and *BCL2L1*, potentially enhancing blast survival and immune evasion (6), in a proposed self-reinforcing circuit rather than an established sequence of events. This feed-forward loop constitutes a central therapeutic vulnerability amenable to combined molecular and immunologic targeting. Arrows, activating signals. AML, acute myeloid leukemia; *BCL2L1*, B-cell lymphoma 2-like protein 1; CCL2, C-C motif chemokine ligand 2; IL, interleukin; IL-10R, IL-10 receptor; OSM, oncostatin M; OSMR, OSM receptor; *SOCS3*, suppressor of cytokine signaling 3; STAT3, signal transducer and activator of transcription 3; TNF-α, tumor necrosis factor-alpha. Created in BioRender. Zangana, K. (2026) https://BioRender.com/4ouolsq, accessed on 21 May 2026.

The convergence of *CEBPA* and *RUNX1* mutations on macrophage-driven STAT3 activation is proposed to constitute a triad of mutant transcription factors, TAMs and STAT3 signaling that could function as a self-reinforcing circuit sustaining AML progression [61]. This circuit is mediated by blast-derived extracellular vesicles and cytokines that remodel the bone marrow into an inflammatory niche, recruiting and polarizing macrophages toward a pro-tumoral M2-like phenotype. Educated TAMs, in turn, produce IL-6 and OSM, resulting in the activation of STAT3 in blasts to enhance their survival, proliferation, and further niche-modifying capacity, while STAT3 signaling within TAMs is proposed to reinforce their M2-like polarization through a positive feedback loop. *CEBPA* mutations confer STAT3 hypersensitivity and a differentiation block, while *RUNX1* mutations impair STAT3 attenuation and reduce *CEBPA* expression. We propose that *CEBPA*- and *RUNX1*-mutant AML converge on a common macrophage-STAT3 axis, a model that, if validated, would represent a disease-sustaining circuit persisting under therapeutic pressure [62], as presented in Figure 4 and Table 1.

The triad consequently extends earlier descriptions of self-reinforcing TAM-STAT3 circuits in two specific ways. First, it places mutation-defined leukemic states upstream of niche remodeling and explicitly distinguishes the blast, macrophage, and stromal compartments instead of treating STAT3 activation as a single tumor cell autonomous event. Second, it converts the synthesis into falsifiable conditional predictions: *RUNX1*-associated convergence is expected to be weaker and more context-dependent than *CEBPA*-associated convergence; OSMR dependence should track stromal OSMR abundance and spatial proximity rather than blast genotype alone; and combined pathway inhibition should be most effective in samples showing concurrent macrophage-state enrichment, cytokine elevation, and blast p-STAT3. These elements are the manuscript’s original integration. They do not establish causality and should not be interpreted as replacing prior TAM-STAT3 models [15].

## 6. Translational Biomarkers and Clinical Implications

### 6.1. Measurable Indicators: Serum OSM and SOCS3, BCL2L1 Expression

Translational biomarkers permit accurate risk classification and dynamic monitoring to inform targeted therapeutic intervention. OSM is a promising, noninvasively measurable biomarker in de novo AML. In a recent retrospective proteomic cohort, serum OSM emerged as a candidate-independent predictor of overall survival, early mortality, and response to induction chemotherapy [32]; this finding awaits prospective, multi-cohort validation.

Clinically, serum OSM has been proposed as a candidate-independent predictor of overall survival, early mortality and induction response, outperforming the European LeukemiaNet (ELN) 2022 risk model. Patients with high OSM levels have substantially lower complete remission rates after both intensive and non-intensive induction. OSMR is principally expressed by bone marrow mesenchymal stromal cells rather than leukemic blasts, consistent with a paracrine paradigm in which OSMR/gp130 heterodimerization, following OSM binding, elicits *JAK1*/*JAK2* cross-phosphorylation and downstream activation of STAT3, PI3K and MAPK cascades that contribute to chemoresistance, survival and disease progression [36]. IL6, SOCS3, and BCL2L1 transcript abundance in AML blasts can be quantified using reverse transcription quantitative PCR (RT-qPCR) [63], providing high sensitivity and specificity for evaluating pathway activation. Elevated IL-6 and dysregulated *SOCS3* reflect perturbed IL-6/STAT3 signaling [64], while increased *BCL2L1* (Bcl-xL) reflects anti-apoptotic activity and treatment resistance [10].

### 6.2. Integration with Mutation Status for Risk Assessment

The cellular source and receiving compartment are decisive for therapeutic interpretation. Current AML data support OSM/OSMR primarily as an outcome-associated blood biomarker and suggest substantial OSMR expression in mesenchymal/stromal cells, whereas direct dependence of primary AML blasts on TAM-derived OSM remains insufficiently demonstrated. A stromal-dominant circuit would predict that OSMR blockade modifies niche support indirectly and may require preserved drug penetration and combination with blast-directed therapy. By contrast, demonstrable blast-intrinsic OSMR expression and OSM-dependent viability would provide a stronger rationale for direct pathway addiction. These alternatives should be resolved using paired marrow plasma, sorted macrophages, stromal cells, and blasts, together with ligand-neutralization and cell-type-specific OSMR perturbation [32,36].

Integrating IL-6/OSM/*SOCS3*/*BCL2L1* biomarker data with conventional mutational profiling (such as *CEBPA* and *RUNX1*) yields a dynamic, functionally informed risk framework that complements static genotypic classification (e.g., European LeukemiaNet 2022). A molecular-functional model would combine the genetic mutation profile as the disease baseline (*CEBPA* and *RUNX1*) with an inflammatory microenvironment readout (IL-6, OSM, *SOCS3* and *BCL2L1*) [65]. Consequently, combining these biomarker axes with mutational context is proposed as a future translational risk-stratification framework, capable in principle of identifying high-risk patients and guiding mechanism-based therapeutic decisions once its component biomarkers have undergone prospective validation, as summarized in Table 2.

### 6.3. Potential Therapeutic Strategies: STAT3 Inhibitors, IL-6/OSMR Blockade, Macrophage Reprogramming

STAT3 represents a central mediator of oncogenic signaling, inflammation, and immune evasion, and its constitutive activation is associated with poor prognosis in multiple cancer types; pharmaceutical STAT3 inhibition is therefore proposed to disrupt tumor-promoting pathways and sensitize tumors to therapy [65]. In AML, several classes of STAT3 inhibitors have been described, targeting distinct points in the pathway: Src homology 2 (SH2) domain inhibitors that prevent STAT3 dimerization; DNA-binding domain inhibitors that block transcriptional activity; upstream JAK2 kinase inhibitors; and indirect agents that affect STAT3 protein stability or post-translational modifications [66]. Small-molecule inhibitors such as nifuroxazide, static and SH-4-54 have reduced tumor cell proliferation and enhanced apoptosis in preclinical cancer models. Combining STAT3 inhibitors with chemotherapy has shown synergistic effects in preclinical bladder-cancer models [67].

Blockade of the IL-6/OSMR signaling axis represents an alternative, microenvironment-directed strategy for inhibiting STAT3 activation in the inflammatory niche proposed to sustain AML. Two therapeutic modalities target this axis: anti-IL-6 monoclonal antibodies that sequester the cytokine ligand, and anti-IL-6 receptor antibodies that block receptor engagement. Tocilizumab, an IL-6 receptor antagonist, blocks both soluble and membrane-bound IL-6R, producing durable inhibition of IL-6-driven JAK/STAT3 signaling and reducing STAT3 phosphorylation through sustained receptor occupancy [68]. Tocilizumab has not been tested in AML directly; evidence that IL-6 blockade favorably modulates outcomes in solid-tumor patients receiving immune checkpoint blockade [69] suggests, by extrapolation, that in the subset of AML patients with IL-6-dependent STAT3 activation, tocilizumab could plausibly disrupt stromal survival signaling and enhance chemosensitivity, a hypothesis that would require dedicated AML clinical investigation. In preclinical ovarian cancer models, the anti-OSMR monoclonal antibody B21 abrogates OSMR-driven STAT3 signaling, reducing cancer cell proliferation and suppressing tumor growth and cisplatin resistance [70]; this agent has not yet been evaluated in AML. Given that OSMR is predominantly expressed on bone-marrow stromal rather than leukemic cells, OSMR blockade in AML would be expected to act on the microenvironment, disrupting paracrine OSM/OSMR-gp130 signaling proposed to sustain blast survival and chemoresistance.

Reprogramming TAMs from a pro-tumor (M2-like phenotype) to an anti-tumor (M1-like phenotype) is a promising therapeutic strategy under active clinical investigation across solid and hematologic cancers [71], with proof-of-concept preclinical support from a murine ascites carcinoma model [72]. TLR agonists, notably TLR4 ligands such as lipopolysaccharide or monophosphoryl lipid A, and TLR7/8 agonists such as imiquimod or resiquimod, engage NF-κB and interferon-regulatory factor (IRF) signaling to drive macrophages toward an M1-like, pro-inflammatory phenotype; imiquimod is FDA-approved as a topical agent for superficial basal cell carcinoma, whereas other TLR agonists remain investigational. Additional strategies described in the solid-tumor macrophage-reprogramming literature include CCL2/CCR2 blockade to inhibit monocyte recruitment, physical TAM depletion, and metabolic reprogramming via phosphoinositide 3-kinase gamma (PI3Kγ) or AMP-activated protein kinase (AMPK) modulation to skew polarization toward M1-like phenotypes [73]. None of these reprogramming strategies has yet been evaluated in AML, and translating them to the bone-marrow niche presents specific challenges: this niche is a distinct, multicellular environment comprising stromal cells, osteoblasts, endothelium, and hematopoietic elements, in which systemic macrophage reprogramming may carry a risk of inflammatory toxicity such as cytokine-release syndrome.

Among the topics discussed here, only OSM/OSMR has AML-cohort data, and these come from a single retrospective proteomic study awaiting prospective validation; it is a prognostic biomarker, not a validated therapeutic target. STAT3 small-molecule inhibitors, IL-6/IL-6R blockade, anti-OSMR antibodies, and macrophage-reprogramming agonists remain, with respect to AML specifically, at the preclinical or early-phase/cross-tumor investigational stage, and should be interpreted as therapeutic hypotheses generated by this review rather than established treatment options.

## 7. Future Perspectives

### 7.1. Need for Multi-Omics Studies Combining Genetics, Cytokines, and Immune Phenotyping

The cellular and molecular heterogeneity of the AML bone-marrow niche mandates a shift from single-modality profiling to integrated, mechanistic multi-omics frameworks. Current prognostic schemas for AML remain dominated by static genomic lesions and cytogenetic aberrations, such as FMS-like tyrosine kinase 3 (*FLT3*) and nucleophosmin 1 (*NPM1*) mutations. Yet these DNA-level readouts alone fail to represent dynamic functional states driven by regulated proteomic and cytokine signaling networks (IL-6/JAK/STAT3, Phosphoinositide 3-Kinase (PI3K)/Protein Kinase B (AKT) and NF-κB) that support blast survival and chemoresistance. Integrative approaches that combine genomic backbones with proteomic and cytokine profiling and deep immune phenotyping are therefore essential to map the dynamic blast-niche interactome underlying therapeutic failure and to reshape how immune function and therapeutic response are understood across cancer systems [74]. Parallel advances in single-cell and spatial technologies now illuminate the spatial organization of immunoregulatory networks within the bone-marrow niche. Integrating single-cell RNA sequencing (scRNA-seq) with spatial transcriptomic platforms (e.g., CosMx and Visium) enables reconstruction of discrete ‘immune neighborhood’ localized clusters of macrophages, stromal populations, T cells, and leukemic blasts responsible for immunosuppressive circuits [75].

The IL-6/OSM/STAT3 axis proposed in this review does not operate in isolation and is best understood as one of several coexisting, and potentially interacting, AML bone-marrow survival programs. The CXCL12/CXCR4 axis retains leukemic and normal hematopoietic progenitors within protective perivascular niches and activates PI3K/AKT and NF-κB signaling in leukemic cells; because NF-κB signaling also governs the pro-inflammatory cytokine output, CXCR4-mediated niche retention may act upstream of, or in parallel with, the cytokine milieu that drives OSM/IL-6-STAT3 engagement, rather than operating through an independent mechanism [76].

Bone-marrow adipocytes and MSCs constitute a second, metabolically distinct survival program, supplying free fatty acids that sustain fatty-acid oxidation-dependent chemoresistance in leukemic blasts; because these same stromal and adipocytic populations are also a principal cellular source of niche IL-6, metabolic support from adipocytes/MSCs and the cytokine-STAT3 axis proposed here likely share a common cellular origin within the niche rather than representing fully separable circuits [77].

IL-1β/NLRP3 inflammasome activation bears the most direct mechanistic relationship to the model proposed in this review: inflammasome-dependent caspase-1 cleavage generates the mature IL-1β already implicated here as part of the *CEBPA*-mutant inflammatory cytokine signature and the broader macrophage-driven feed-forward loop, positioning NLRP3 inflammasome activity as a plausible upstream contributor to, rather than a competing alternative to, IL-6/OSM-driven STAT3 signaling in the AML niche [78].

Direct experimental dissection of whether these programs are convergent, compensatory, or independently sufficient for leukemic survival has not, to our knowledge, been undertaken in AML and represents an important direction for future work; from a therapeutic standpoint, however, the possibility of convergence or compensation is itself actionable, since agents targeting the IL-6/OSM/STAT3 axis in isolation may prove insufficient if CXCR4-, adipocyte/MSC-, or inflammasome-driven survival signaling remains active in parallel.

Recent AML single-cell studies further argue for replacing bulk TAM abundance with state-resolved and spatial measurements. Macrophage subpopulations associated with non-complete remission show distinct FCAR/FCGR3A and metabolic programs [27], while contemporary single-cell reviews emphasize that blast, immune, and stromal states cannot be inferred reliably from bulk profiles [79]. Therapeutically, colony-stimulating factor 1 receptor (CSF1R), PI3Kγ, MER proto-oncogene tyrosine kinase (MerTK), AXL receptor tyrosine kinase (Axl), triggering receptor expressed on myeloid cells 2 (TREM2), leukocyte immunoglobulin-like receptor B (LILRB), V-domain immunoglobulin suppressor of T-cell activation (VISTA), and CD40 are increasingly discussed as myeloid-reprogramming or myeloid-checkpoint targets, but most evidence remains cross-tumor or preclinical rather than AML-specific [80]. In AML, checkpoint-directed strategies should therefore be framed as subtype- and biomarker-dependent experiments, not as established extensions of the proposed triad. These developments strengthen the rationale for measuring macrophage state and spatial context while also underscoring the present translational gap.

### 7.2. Validation of OSMR and STAT3 Activity as Predictive Tools

Although serum OSMR expression and phosphorylation of intracellular STAT3 have surfaced as promising biomarkers, their implementation in clinical practice mandates stringent validation. The designation of OSMR as a routine, evidence-based biomarker will require large-scale, prospective validation in independent patient cohorts comprising multi-ethnic populations and heterogeneous treatment regimens, together with standardized assay methodology and predefined performance thresholds to demonstrate clinical relevance and reliability [32].

Establishing the clinical validity of inflammatory biomarkers will also require rigorous standardization of analytical procedures. In contrast to categorical genetic lesions, these biomarkers function as continuous quantitative variables and therefore demand precisely defined, clinically validated threshold values. This issue is particularly pertinent for STAT3, as phospho-STAT3 measurements alone offer an incomplete assessment of pathway activity and exhibit substantial variability across cytogenetic subgroups; phospho-STAT3 is typically elevated in normal-karyotype AML while infrequent in core-binding factor leukemias [64]. The expression levels of canonical STAT3-responsive genes, including *BCL2L1*, *SOCS3*, and *ICAM1*, provide a more functionally robust indicator of pathway activation than static protein abundance [81]. Developing standardized assays, establishing analytical performance characteristics, and defining clinically actionable cut-points will be essential steps toward integrating these biomarkers into routine diagnostic practice. Additionally, integrating inflammatory biomarkers into established minimal residual disease (MRD) frameworks represents an essential translational advance. Rather than conceptualizing mutation clearance and inflammatory resolution as discrete pathways, future prognostic models should quantify an “inflammatory residual disease” state, defined by IL-6/OSMR-driven microenvironmental activation despite morphological or molecular remission as a biologically grounded predictor of early relapse [82].

### 7.3. Exploring Combined Immuno-Targeted Therapies

The future of cancer therapy lies in rationally designed combinations that target both oncogenic pathways and the immune microenvironment. Combining immune checkpoint inhibitors (ICIs) with targeted therapies such as kinase inhibitors, anti-angiogenic agents, or OSMR/STAT3 pathway inhibitors has shown synergistic effects in clinical studies of solid tumors [83]. The redundancy of cytokine signaling networks in AML suggests that monotherapies targeting the IL-6/STAT3 axis may be insufficient, necessitating the exploration of rational combination strategies. A promising avenue lies in the dual targeting of inflammatory pathways and immune checkpoints. Preclinical data in AML cells indicate that STAT3 signaling facilitates immune evasion and is associated with upregulation of PD-L1 expression [84]. Consequently, combining STAT3 inhibition with PD-1/PD-L1 blockade could theoretically release the brakes on T-cell immunity while simultaneously sensitizing leukemic blasts to immune surveillance.

**Figure 5:**
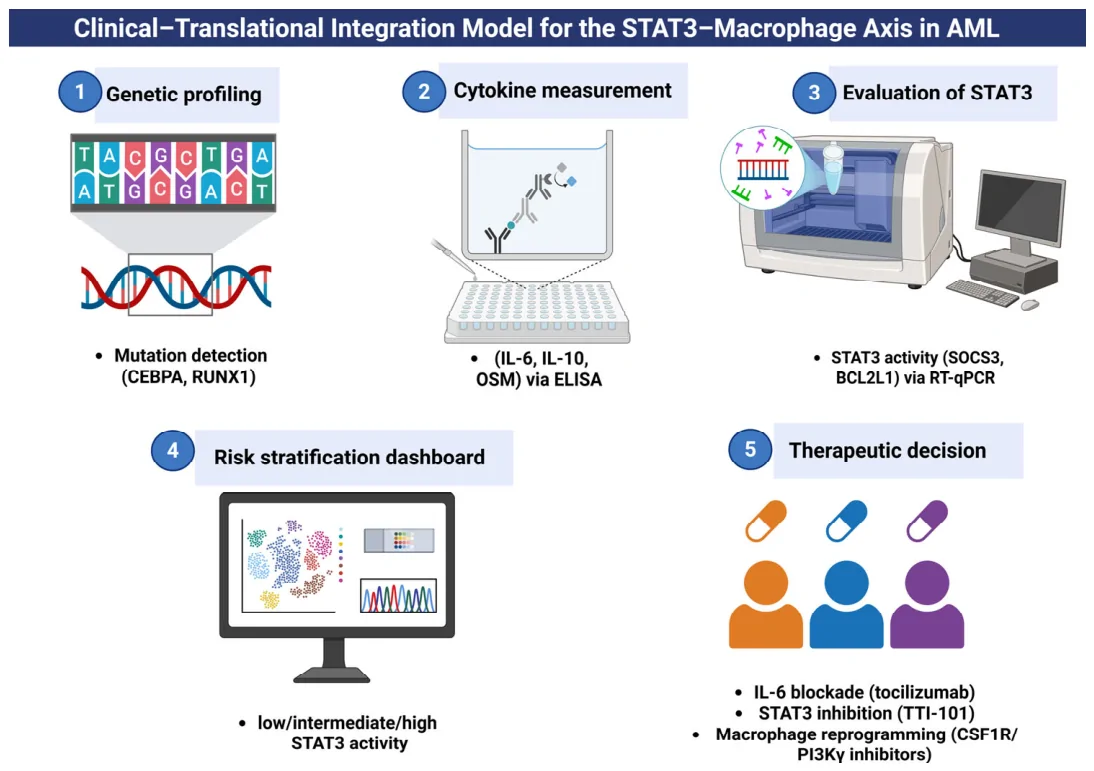
Proposed hypothesis-generating conceptual framework integrating genetic, cytokine, and STAT3 pathway profiling into a clinical-translational workflow for biomarker-guided management of AML. (1) Molecular genetic profiling detects *CEBPA* and *RUNX1* mutations to establish baseline genomic risk. (2) Serum cytokine quantification of IL-6, IL-10, and OSM by ELISA defines the inflammatory microenvironmental state. (3) RT-qPCR measurement of *SOCS3* and *BCL2L1* transcripts in bone marrow blasts evaluates downstream STAT3 pathway activation. (4) Integrated multi-dimensional data are used to stratify patients into low, intermediate, or high STAT3 activity risk categories. (5) The proposed risk categories could, after prospective validation, inform investigation of mechanism-based therapeutic strategies, including IL-6 blockade, STAT3 inhibition, or macrophage-directed approaches. AML, acute myeloid leukemia; *BCL2L1*, B-cell lymphoma 2-like protein 1; CSF1R, colony-stimulating factor 1 receptor; ELISA, enzyme-linked immunosorbent assay; IL, interleukin; OSM, oncostatin M; PI3Kγ, phosphoinositide 3-kinase gamma; RT-qPCR, reverse transcription quantitative PCR; *SOCS3*, suppressor of cytokine signaling 3; STAT3, signal transducer and activator of transcription 3; TTI-101, STAT3 small-molecule inhibitor. Created in BioRender. Zangana, K. (2026) https://BioRender.com/20vmzj2, accessed on 21 May 2026.

Integrated targeted agents with immune checkpoint inhibitors are supported by mechanistic and translational data, in which targeted drugs can remodel the tumor microenvironment and enhance neoantigen exposure and promote effector-cell infiltration, thereby potentiating ICI activity [85]. Cytokine-driven activation of the OSMR-STAT3 signaling axis has been shown to mediate resistance to chemotherapy in ovarian cancer with antibody blockade of OSMR restoring chemosensitivity [70], while IL-6 blockade has separately been shown to favorably modulate outcomes in cancer patients receiving immune checkpoint blockade [69]; whether concurrent OSMR/IL-6-STAT3 pathway blockade would similarly restore treatment sensitivity in AML has not been verified. Ultimately, clinical development across solid and hematologic malignancies is increasingly focused on biomarker-driven trials evaluating rational combinations of immune checkpoint inhibitors with targeted microenvironmental or kinase inhibitors to test synergistic efficacy [86], as depicted in Figure 5 and detailed in Table 3.

### 7.4. Complexity and Heterogeneity of STAT3 Signaling in AML Immunity

The evidence reviewed above emphasizes a pro-leukemic, immunosuppressive function for STAT3 within the AML niche; however, this framing does not capture the full immunological complexity of STAT3 signaling in AML. STAT3 activity intrinsic to leukemic blasts has also been reported to be required for their recognition and elimination by natural killer (NK) cells, such that STAT3 loss impaired, rather than enhanced, NK-cell-mediated surveillance of leukemic cells [81]. This finding is not readily accommodated by a model in which STAT3 activation uniformly favors immune evasion, and instead indicates that the net immunological consequence of STAT3 signaling is compartment-dependent, varying with the cellular source of STAT3 activity (blast-intrinsic versus macrophage-intrinsic), the immune effector population under consideration, and disease stage. A comparable context-dependence has already been noted for IL-10 within the same niche: IL-10/STAT3 signaling in macrophages and regulatory T cells reinforces immunosuppression, whereas IL-10 can directly restrain AML blast proliferation and has been associated with improved survival in some cohorts. Taken together, these observations argue against treating STAT3 pathway activation as a uniformly unfavorable biological readout and instead support a compartment- and context-specific interpretive framework.

STAT3 pathway engagement is similarly unlikely to be uniform across AML molecular and cytogenetic subtypes. Phospho-STAT3 positivity itself varies by cytogenetic subgroup, being frequently detected in normal-karyotype AML but comparatively infrequent in core-binding-factor leukemias [64]. Disease ontogeny appears to contribute an additional layer of heterogeneity: bone marrow mesenchymal stromal cells from de novo AML display broader cytokine dysregulation (elevated IL-1β, IL-6, IL-32, VEGFA, and CXCL12) than those from secondary AML with myelodysplasia-related changes, in which the increase is largely restricted to IL-6 [54]. Genetic risk and inflammatory cytokine burden may also dissociate, as illustrated by *CEBPA*-mutant AML in Table 1, where an otherwise favorable intrinsic genetic risk profile may be offset by a high cytokine burden driving inflammation-dependent relapse. Collectively, this heterogeneity suggests that the proposed Mutation–Macrophage–STAT3 axis is unlikely to apply uniformly across AML and may instead be most relevant to specific molecular or cytogenetic subgroups, a distinction that should be tested directly in subtype-stratified cohorts rather than assumed.

### 7.5. A Testable Translational Roadmap for the Proposed Axis

A first validation study should enroll newly diagnosed adults prospectively and stratify them by *CEBPA* alteration, *RUNX1* alteration, AML ontogeny, and ELN risk, with a parallel validation cohort receiving the same treatment backbone. At diagnosis, day 14, remission assessment, and relapse, paired marrow and blood should undergo cytokine profiling, phospho-flow for blast and myeloid p-STAT3, scRNA-seq/cellular Indexing of Transcriptomes and Epitopes by Sequencing (CITE-seq), and spatial profiling of OSM/OSMR-expressing macrophage–stromal–blast neighborhoods. The prespecified primary mechanistic endpoint should be concordance between macrophage-state abundance, stromal or blast OSMR localization, and compartment-specific STAT3 activity. Secondary endpoints should include complete remission, measurable residual disease clearance, event-free survival, and treatment-related inflammatory toxicity [75,97].

A subsequent biomarker-enriched window-of-opportunity study could recruit patients with high OSM/IL-6, an OSMR-positive stromal niche, and elevated blast p-STAT3. Short exposure to a pathway-directed agent before standard therapy would test pharmacodynamic suppression of p-STAT3 and downstream *BCL2L1* without initially relying on response rate as proof of mechanism. Randomization to standard therapy with or without the investigational agent should occur only after demonstrating target engagement. Safety monitoring should prioritize infection, cytopenias, hepatic toxicity, impaired normal myelopoiesis, and cytokine-release or rebound inflammation. Because broad macrophage depletion may damage normal marrow defense and repair, state-selective reprogramming or temporally limited blockade may offer a more feasible strategy than continuous pan-macrophage suppression [98,99].

## 8. Conclusions

This review proposes the Mutation–Macrophage–STAT3 axis as an emerging conceptual model of AML pathogenesis, in which *CEBPA* and *RUNX1* mutations, despite divergent prognostic weight, are each independently associated with overproduction of IL-1β, TNF-α, IL-6, and CCL2, a pattern consistent with remodeling of the bone marrow into a chronically inflamed niche. CCL2-recruited monocytes are proposed to polarize toward M2-like tumor-associated macrophages that reciprocally produce OSM and IL-6, a process that, if confirmed, would activate gp130/JAK-dependent STAT3 phosphorylation in leukemic blasts and upregulate *BCL2L1* and *SOCS3*, contributing to survival and chemoresistance. IL-10 from macrophages and stroma has additionally been reported, in related contexts, to further activate STAT3 and suppress antigen-presenting cell function and cytotoxic T-cell responses, suggesting a potential self-reinforcing circuit of immune evasion that we propose may operate largely independent of the initiating genetic lesion. Translationally, integrating mutational profiling with serum OSM and IL-10 quantification alongside RT-qPCR measurement of *SOCS3* and *BCL2L1* transcripts generates a dynamic risk framework, positioning aberrant inflammation as a candidate prognostic determinant and a hypothesis-generating target for IL-6/OSMR/STAT3-directed or macrophage-reprogramming therapies in selected AML subtypes.

## Figures and Tables

**Table 1:** Comparative immunomodulatory profile and STAT3 activation in *CEBPA*- versus *RUNX1*-mutant AML.

Genetic Lesion	Pathogenic Cytokine Signature	Tumor Microenvironment Response (Macrophage Phenotype)	Downstream STAT3 Pathway Activation	Clinical Correlation and Mechanistic Implication
*CEBPA* mutation	↑ IL-1β, TNF-α (pro-inflammatory); ↑ CCL2 (chemotactic)	Proposed monocyte recruitment and M2-like skewing; hypothesized OSM secretion fueling paracrine STAT3 activation	Proposed STAT3 activation; hypothesized increases in downstream STAT3-responsive targets, including SOCS3 and BCL2L1	Favorable genetic risk overall; hypothesized that high cytokine burden may contribute to inflammation-dependent relapse risk
*RUNX1* mutation	↑ IL-6, IL-1β, TNF-α (pro-inflammatory and autocrine)	Hypothesized M2-like skewing; candidate increase in *OSMR* expression and IL-10 secretion	Hypothesized STAT3 pathway activation; direct evidence linking *RUNX1* mutation to elevated p-STAT3 and downstream targets in AML remains limited	Adverse prognosis associated with *RUNX1*-mutant AML; whether STAT3 activation contributes mechanistically to this outcome remains unestablished.
Convergent outcome	Inflammatory cytokine overproduction	Recruitment and polarization of pro-tumorigenic macrophages	Persistent STAT3 pathway activation	Aberrant inflammation as a shared therapeutic vulnerability, despite divergent genetic risk

AML, acute myeloid leukemia; *BCL2L1*, B-cell lymphoma 2-like protein 1; CCL2, C-C motif chemokine ligand 2; IL, interleukin; *IL-6*, interleukin-6 gene; OSM, oncostatin M; *OSMR*, OSM receptor gene; p-STAT3, phosphorylated STAT3; *SOCS3*, suppressor of cytokine signaling 3; STAT3, signal transducer and activator of transcription 3; TNF-α, tumor necrosis factor-alpha. ↑ indicates increased expression or activity.

**Table 2:** Soluble and transcriptional biomarkers of the inflammatory STAT3 axis in AML.

Biomarker	Detection Method	Biological Role in the Pathway	Clinical Interpretation
IL-6, IL-10, and OSM	Serum/plasma ELISA	Primary upstream cytokines driving JAK/STAT3 activation in leukemic blasts and microenvironmental cells	Elevated concentrations indicate altered inflammatory signaling; prognostic interpretation is analyte- and context-dependent, particularly for IL-10
Soluble OSMR	Serum/plasma ELISA (circulating); RT-qPCR/flow cytometry (cellular)	Candidate circulating marker of OSM/OSMR pathway activity; cellular source and link to macrophage state require further validation	Elevated circulating sOSMR has been associated with adverse clinical outcomes in AML; its relationship with the immunosuppressive tumor microenvironment requires further validation
*SOCS3* mRNA	RT-qPCR (bone marrow blasts)	Immediate-early *STAT3* transcriptional target; persistent overexpression indicates failed negative feedback inhibition of JAK signaling	Transcriptional signature of sustained STAT3 activation and evidence of failed negative-feedback attenuation
*BCL2L1* mRNA	RT-qPCR (bone marrow blasts)	Key anti-apoptotic effector gene under direct *STAT3* transcriptional control; encodes Bcl-xL, which blocks programmed cell death	Elevated expression reflects a STAT3-driven survival advantage and intrinsic resistance to chemotherapy

AML, acute myeloid leukemia; *BCL2L1*, B-cell lymphoma 2-like protein 1; Bcl-xL, B-cell lymphoma extra-large; ELISA, enzyme-linked immunosorbent assay; IL, interleukin; JAK, Janus kinase; mRNA, messenger RNA; OSM, oncostatin M; OSMR, OSM receptor; RT-qPCR, reverse transcription quantitative polymerase chain reaction; *SOCS3*, suppressor of cytokine signaling 3; STAT3, signal transducer and activator of transcription 3. Note: OSMR appears to be expressed predominantly by bone marrow stromal/mesenchymal cells rather than leukemic blasts, supporting a primarily paracrine microenvironmental role.

**Table 3:** Previous Studies on the Roles of *RUNX1*, *CEBPA*, IL-10/OSM in AML.

	Objective	Outcome	Conclusions	Reference
1	To determine if *CEBPA* mutations are causative for AML development in the context of *RUNX1*/*SRSF2* mutations using patient-derived iPSCs.	Introduction of a *CEBPA* bZIP domain mutation on a *RUNX1*/*SRSF2* background promoted disease progression by blocking myeloid differentiation and increasing progenitor self-renewal.	Mutant *CEBPA* is causative for MDS progression toward AML, especially when co-occurring with *RUNX1* mutations.	[87]
2	To examine how t(8;21) (*RUNX1::ETO*) LSCs activate signaling pathways to promote growth and relapse.	t(8;21) LSCs aberrantly activate VEGF and IL-5 pathways via a circuit involving *RUNX1::ETO*, *AP-1*, and *GATA2*; *CEBPA* double mutant AML activates alternative cytokine receptors.	Aberrant pathway activation by *RUNX1*/*CEBPA* mutations supports LSC survival and relapse risk in AML subtypes.	[48]
3	To study clonal evolution during CMML progression to secondary AML focusing on transcription factor gene dynamics (including *RUNX1*/*CEBPA*).	*RUNX1* mutations often arose as late events during transformation; acquisition of cytogenetic changes or additional TF gene mutations (including *CEBPA*) marked disease progression risk.	Dynamic changes in TF genes like *RUNX1*/*CEBPA* are critical for leukemic transformation risk stratification.	[88]
4	To summarize updates in diagnosis, risk stratification, and management of AML, including the role of molecular markers like *RUNX1*/*CEBPA*.	New biological insights have led to improved classification systems incorporating molecular findings such as *RUNX1*/*CEBPA* status for prognosis and therapy selection.	Integration of molecular data (including *RUNX1*/*CEBPA*) is essential for personalized AML management strategies.	[89]
5	To elucidate genetic and transcriptomic alterations in AML through whole-exome sequencing and transcriptome analysis, focusing on regulatory networks involving *RUNX1*/*CEBPA*.	Key transcription factors including *RUNX1* and *CEBPA* were frequently mutated/dysregulated, affecting cell cycle, differentiation, apoptosis, and immune regulation pathways in AML cells.	Disruptions in regulatory networks involving *RUNX1*/*CEBPA* contribute to AML pathogenesis and offer potential therapeutic targets.	[90]
6	To investigate synergy between ziftomenib (menin inhibitor) and selinexor (nuclear export inhibitor) in *NPM1*-mutant AML models affecting PU.1–*CEBPA*/*RUNX* axis function.	Combination therapy synergistically inhibited growth of *NPM1*-mutant AML cells by restoring nuclear localization of *NPM1c* with PU.1–CEBPA/RUNX axis activation, leading to differentiation/apoptosis without toxicity to normal cells.	Targeting the PU.1–CEBPA/*RUNX* axis via combination therapy may be an effective strategy for *NPM1*-mutant AML treatment beyond *KMT2A*-r models.	[91]
7	To investigate clinical/molecular features of *TP53*-mutant AML, including the impact of *CEBPA*/*RUNX* alterations on prognosis/outcomes.	*TP53*-mutant patients with CEBPA bZIP mutations or *RUNX1::RUNX1T1* fusion had better survival; isolated *RUNX1* mutation was associated with worse prognosis among *TP53*-mutants.	Molecular context (e.g., co-mutations with *CEBPA*/*RUNX* family genes) significantly influences prognosis in *TP53*-mutant AML patients; personalized approaches needed based on mutational landscape.	[92]
8	To map chromatin accessibility/regulatory networks across genetically defined subtypes including those defined by double-mutant *CEBPA* or *RUNX1::RUNX1T1* fusions.	Distinct epigenomic/transcriptomic clusters corresponded to genetic subtypes (e.g., dmCEBPA, *RUNX1::RUNX1T1*), reflecting unique regulatory mechanisms affecting differentiation blockades.	Subtype-specific regulatory networks driven by *CEBPA*/*RUNX1T1* define heterogeneity in AML; these may inform targeted therapies.	[93]
9	To analyze how ELN 2017/2022 genetic risk classifications perform among older/unfit patients treated with venetoclax-azacitidine, focusing on outcomes by mutational status including *RUNX1* mutations.	ELN criteria poorly discriminated outcomes after venetoclax-azacitidine; however, presence of certain mutations (e.g., favorable outcomes seen with some cases harboring *NPM1* or *RUNX1* mutations).	New molecular signatures beyond current ELN criteria are needed to guide therapy selection among older/unfit patients; mutational context matters.	[94]
10	To review frontline/later-line therapies and research directions in modern AML management—including roles for targeted therapies against molecular drivers like *CEBPA*/*RUNX1::RUNX1T1*.	Since 2017, multiple targeted agents have been approved or investigated based on underlying genetics such as *FLT3*/*NPM1*/*IDH*/*CBF* fusions/double-mutant CEBPA, shifting standard-of-care paradigms.	Ongoing research into novel agents targeting specific genetic drivers will continue to reshape induction and consolidation strategies; precision medicine is central.	[95]
11	To review cytokine networks in AML, focusing on IL-10’s dual role in disease progression and immune modulation.	High plasma IL-10 correlates with better survival/remission; IL-10 inhibits AML blast proliferation but may also support leukemic stem cell (LSC) survival via immunosuppression.	IL-10 has context-dependent effects: it can suppress AML blasts but also create an immunosuppressive niche supporting LSCs; targeting IL-10 signaling could enhance chemotherapy efficacy.	[37]
12	To investigate IL-10 receptor (IL-10R) as a therapeutic target for CAR-T cell therapy in AML.	IL-10R is overexpressed on most AML cells; CAR-T cells targeting IL-10R show potent cytotoxicity against AML in vitro/in vivo with minimal off-target effects.	IL-10R is a promising immunotherapy target for AML; ligand-based CAR-T therapy may improve prognosis.	[96]
13	To summarize inflammatory cytokine signaling in AML, including anti-inflammatory mediators like IL-10.	Elevated IL-10 was detected in AML patients; associated with both improved survival and increased immunosuppression by bone marrow stromal cells.	The role of IL-10 is complex—while it can inhibit leukemic growth, it may also foster immune escape; understanding this balance is key for new therapies.	[38]

AML, acute myeloid leukemia; CAR-T, chimeric antigen receptor T-cell; CBF, core-binding factor; CEBPA, CCAAT/enhancer-binding protein alpha; CMML, chronic myelomonocytic leukemia; ELN, European LeukemiaNet; FLT3, Fms-like tyrosine kinase 3; GATA2, GATA-binding protein 2; IDH, isocitrate dehydrogenase; IL, interleukin; IL-10R, interleukin-10 receptor; iPSC, induced pluripotent stem cell; KMT2A-r, KMT2A-rearranged; LSC, leukemic stem cell; MDS, myelodysplastic syndrome; NPM1, nucleophosmin 1; OSM, oncostatin M; PU.1, Spi-1 proto-oncogene transcription factor; RUNX1, runt-related transcription factor 1; RUNX1::ETO (RUNX1::RUNX1T1), RUNX1-RUNX1T1 fusion protein; SRSF2, serine and arginine rich splicing factor 2; TF, transcription factor; TP53, tumor protein p53; VEGF, vascular endothelial growth factor. Note: This table summarizes representative studies describing the biological, prognostic, and therapeutic relevance of RUNX1, CEBPA, and IL-10/OSM-associated signaling in AML. Findings should be interpreted within the context of study design, patient population, molecular subtype, and disease stage, as the clinical impact of these biomarkers may vary across AML subgroups.

## Data Availability

No new data were created or analyzed in this study. Data sharing is not applicable to this article.

## Abbreviations

The following abbreviations are used in this manuscript:

AKT	Protein kinase B
AML	Acute myeloid leukemia
AMPK	AMP-activated protein kinase
AP-1	Activator protein 1
*ARG1*	Arginase 1
*BCL2*	B-cell lymphoma 2
*BCL2L1*	BCL2-like 1 (gene; encodes Bcl-xL)
Bcl-xL	B-cell lymphoma-extra large
BM-MSC	Bone marrow mesenchymal stromal cell
BMM	Bone marrow microenvironment
bZIP	Basic leucine zipper
CAR-T	Chimeric antigen receptor T cell
CBF	Core-binding factor
CBFβ	Core-binding factor beta
CCL2	C-C motif chemokine ligand 2
CCR2	C-C chemokine receptor type 2
CD8	Cluster of differentiation 8
CD34	Cluster of differentiation 34
CD40	Cluster of differentiation 40
CD163	Cluster of differentiation 163
CD206	Cluster of differentiation 206
C/EBPα	CCAAT/enhancer-binding protein alpha (protein)
*CEBPA*	CCAAT/enhancer-binding protein alpha (gene)
C/EBPβ	CCAAT/enhancer-binding protein beta
CHIP	Clonal hematopoiesis of indeterminate potential
CITE-seq	Cellular indexing of transcriptomes and epitopes by sequencing
CMML	Chronic myelomonocytic leukemia
CSF1R	Colony-stimulating factor 1 receptor
*CSF3R*	Colony-stimulating factor 3 receptor
CXCL12	C-X-C motif chemokine ligand 12
CXCR4	C-X-C chemokine receptor type 4
DBD	DNA-binding domain
*E2F2*	E2F transcription factor 2
ELISA	Enzyme-linked immunosorbent assay
ELN	European LeukemiaNet
ERK1/2	Extracellular signal-regulated kinases 1 and 2
FCAR	Fc alpha receptor (CD89)
FCGR3A	Fc gamma receptor IIIA (CD16A)
FDA	Food and Drug Administration
*FLT3*	FMS-like tyrosine kinase 3
G-CSF	Granulocyte colony-stimulating factor
*GATA2*	GATA-binding protein 2
gp130	Glycoprotein 130
HIV	Human immunodeficiency virus
HSC	Hematopoietic stem cell
*ICAM1*	Intercellular adhesion molecule 1
ICI	Immune checkpoint inhibitor
IDH	Isocitrate dehydrogenase
IFN-γ	Interferon gamma
IKK	IκB kinase
IL	Interleukin
IL-1β	Interleukin-1 beta
IL-5	Interleukin-5
IL-6	Interleukin-6
IL-6R	Interleukin-6 receptor
IL-10	Interleukin-10
IL-10R	Interleukin-10 receptor
IL-12	Interleukin-12
IL-32	Interleukin-32
IκBα	Inhibitor of kappa B alpha
iPSC	Induced pluripotent stem cell
IRF	Interferon-regulatory factor
JAK	Janus kinase
JAK1	Janus kinase 1
JAK2	Janus kinase 2
KIR	Kinase inhibitory region
LILRB	Leukocyte immunoglobulin-like receptor B
LPS	Lipopolysaccharide
LSC	Leukemic stem cell
M1-like	Classically activated macrophage state
M2-like	Alternatively activated macrophage state
MAPK	Mitogen-activated protein kinase
*MCL1*	Myeloid cell leukemia 1
MDS	Myelodysplastic syndrome
MDSC	Myeloid-derived suppressor cell
MerTK	MER proto-oncogene tyrosine kinase
MHC	Major histocompatibility complex
miR-9	MicroRNA-9
MPN	Myeloproliferative neoplasm
mRNA	Messenger RNA
MRD	Measurable residual disease
MSC	Mesenchymal stromal cell
MyD88	Myeloid differentiation primary response 88
NF-κB	Nuclear factor kappa B
NK	Natural killer cell
NLRP3	NOD-, LRR- and pyrin domain-containing protein 3
*NPM1*	Nucleophosmin 1
OSM	Oncostatin M
OSMR	Oncostatin M receptor
PD-1	Programmed cell death protein 1
PD-L1	Programmed death-ligand 1
PDGF-BB	Platelet-derived growth factor-BB
PI3K	Phosphoinositide 3-kinase
PI3Kγ	Phosphoinositide 3-kinase gamma
p-STAT3	Phosphorylated STAT3
PU.1	PU.1 transcription factor
RT-qPCR	Reverse transcription quantitative polymerase chain reaction
*RUNX1*	Runt-related transcription factor 1
scRNA-seq	Single-cell RNA sequencing
SH2	Src homology 2
*SOCS3*	Suppressor of cytokine signaling 3
sOSMR	Soluble oncostatin M receptor
STAT1	Signal transducer and activator of transcription 1
STAT2	Signal transducer and activator of transcription 2
STAT3	Signal transducer and activator of transcription 3
TAM	Tumor-associated macrophage
TGF-β	Transforming growth factor beta
TIRAP	Toll/interleukin-1 receptor domain-containing adaptor protein
TLR	Toll-like receptor
TME	Tumor microenvironment
TNF-α	Tumor necrosis factor alpha
*TP53*	Tumor protein p53
TREM2	Triggering receptor expressed on myeloid cells 2
VEGF	Vascular endothelial growth factor
VEGFA	Vascular endothelial growth factor A
VISTA	V-domain immunoglobulin suppressor of T-cell activation
